# Treatment of Uterine Fibroids with Galvanism by Profound Puncture

**Published:** 1878-10-20

**Authors:** 


### TREATMENT OF UTERINE FIBROIDS WITH GALVANISM BY PROFOUND PUNCTURE

---

Is the title of a most valuable article in the July number, 1878, of the *American Journal of Medical Sciences* by Drs. Gilman Kimball and Emphraim Cutter. The paper is the one which was substantially, if not verbally, offered at the recent session of the American Medical Association. The treatment by the plan indicated in the article was begun in 1871 Of the 50 cases treated since that time, in seven cases, the growth was not arrested; four died; in thirty-two cases the growth was arrested; three cases were relieved and four cases cured.

The battery used by these gentlemen consists of eight plates each of carbon and zinc, 9 by 6 inches. The first four pairs are arranged zinc and carbon; the remaining four carbon and zinc. They are pierced with three circular holes, arranged triangularly—two at the top, and one in the centre, below. Cylinders of hard rubber run through and secure the plates in position by means of nuts. The conductors are made of copper, and are properly insulated; at their extremities are the electrodes. The solution used in the battery is made by the following formula:—R. Pottassium bichromate, saturated solution, Oj; sulphuric acid,

℥vij. Quantity of current is what is required, and it must be profoundly applied, and galvanic action must be confined to the tumor alone.

The zinc electrode comes away readily after the operation, but the carbon electrode sticks in the tissues This is cited as an evidence of the passage of the galvanic current through the electrodes.

The duration of the application of the electrodes has varied from three to fifteen minutes. In each individual case the applications have varied in number from one to nineteen, at intervals of from seven to fourteen days.

The electrodes may be introduced directly through the abdominal, or the vaginal, or the rectal walls, into the fibroid.

Of the fifty cases, only three seem to have been negresses.—"Virginia Medical Monthly."

